# AI-supported visual-semantic word teaching from the perspective of educational psychology: instant gains in Turkish acquisition, computational choice, and learners’ psychological experience

**DOI:** 10.3389/fpsyg.2026.1906982

**Published:** 2026-09-03

**Authors:** Erçin Ayhan

**Affiliations:** Department of Teaching Turkish as a Foreign Language, Institute of Turkish Studies, Hacettepe University, Ankara, Türkiye

**Keywords:** teaching Turkish as a foreign/second language, artificial intelligence, vocabulary acquisition, visual-semantic coherence, AI-generated images, computer-assisted language learning, self-determination theory, quasi-experimental design

## Abstract

**Background:**

Teaching Turkish to A1–A2 learners relies heavily on word selection and multimodal presentation. While traditional curricula prioritize frequency-based lists, they often underestimate the visual-semantic dimension critical for novice learners. Computational tools such as Word2Vec and CLIP offer a data-driven alternative by quantifying word–image alignment, yet their pedagogical efficacy in authentic classrooms remains empirically underexamined. Furthermore, the comparative effectiveness of AI-generated versus authentic photographs in vocabulary instruction constitutes an unresolved issue in computer-assisted language learning research.

**Aim:**

This pilot study investigated whether high visual-semantic coherence, computed using CLIP and Word2Vec, enhances immediate vocabulary gains compared with low-coherence lists. It further examined the non-inferiority of AI-generated images relative to real photographs and explored the educational-psychological mechanisms—motivation, self-regulation, cognitive load, and achievement goals—that underlie stakeholders’ experiences. Phase 2 was designed to explain and contextualize Phase 1 results by identifying the psychological mechanisms underlying observed learning outcomes, thereby achieving integration through the connecting strategy.

**Materials and methods:**

An explanatory sequential mixed-methods design was employed. Phase one assigned 60 A1–A2 learners to three quasi-experimental conditions: (a) high-CLIP words with AI-generated images (*n* = 15), (b) high-CLIP words with real photographs (*n* = 15), and (c) low-CLIP words with textbook images (*n* = 30). The use of non-equivalent word sets across conditions constrains causal interpretation. Vocabulary knowledge was assessed via the Vocabulary Knowledge Scale at pretest, post-test, and week four, though the absence of delayed testing limits conclusions to immediate learning. Phase two administered validated instruments—TAM, MSLQ, AGQ, Paas cognitive load scale, and SDT basic needs scale—to 60 students and 15 instructors.

**Results:**

Both experimental conditions significantly outperformed the control (*p* < 0.001, *d* = 1.42). No significant difference emerged between AI and real images (*p* = 0.678), though the small per-group sample (*n* = 15) provided adequate power only for large effects (*d* ≥ 0.75). Students reported moderate motivation (*M* = 3.52) and perceived fairness (*M* = 3.61), whereas instructors exhibited lower motivation (*M* = 2.89, *p* = 0.005) and higher perceived effort (*M* = 3.67 vs. 2.94, *p* = 0.003). Mastery-approach goals correlated positively with vocabulary gains (*r* = 0.48, *p* < 0.01). Cognitive load was moderate (*M* = 4.2/9) and uniform across conditions. Basic-needs satisfaction significantly predicted intrinsic motivation (*β* = 0.62, *p* < 0.001).

**Conclusion:**

AI-driven visual-semantic word selection demonstrates preliminary promise for concrete vocabulary instruction. Nevertheless, the quasi-experimental design, confounding word sets, absence of delayed post-tests, and limited statistical power necessitate cautious interpretation. Replication with standardized word pools, delayed assessments, and objective proficiency measures is essential to establish causal efficacy and to determine whether AI-generated images offer genuine equivalence—or merely undetected non-inferiority—relative to authentic photographs. The non-significant difference between AI-generated and authentic photographs should be interpreted as the absence of evidence for a difference, not as evidence of absence of difference.

## 1 Introduction

When teaching Turkish as a foreign language, word choice and presentation style play an essential role for beginners (A1–A2). Traditional methods often focus on commonly used words; however, this approach may underestimate the essential visual dimension of words for novice learners who benefit substantially from concrete visual referents (Huang et al., 2023). Turkish is an agglutinative language characterized by productive suffixation: a single verb root can generate over 2,000 inflected forms, and derivational morphemes exceed 100 in number (Hankamer, 1989; Göksel and Kerslake, 2005). This morphological richness creates a substantial vocabulary burden for learners, as the number of possible word forms far exceeds what can be efficiently stored through rote memorization alone. Consequently, computational tools that identify semantically coherent, visually salient word sets become particularly valuable for Turkish vocabulary instruction, where principled selection can reduce the cognitive load associated with morphological decomposition (Sarıtaş et al., 2024).

Huang et al. (2023), in their bibliometric analysis of 516 AI-enhanced language education studies (2000–2019), identified vocabulary learning as the sixth most popular research topic and emphasized that natural language processing tools were frequently applied to develop personalized vocabulary learning systems. Their review highlighted the need for more empirical classroom-based studies that move beyond theoretical discussions of AI’s advantages to rigorously evaluate its pedagogical effectiveness—a gap the present pilot study addresses.

In recent years, distributed semantic models such as Word2Vec and multimodal text-visual alignment models such as CLIP have enabled us to compute visual and semantic associations for words (Radford et al., 2021; Sarıtaş et al., 2024). Word2Vec (Mikolov et al., 2013) is a neural network-based approach that represents words as dense vectors in a continuous semantic space. The Continuous Bag-of-Words (CBOW) architecture predicts a target word from its surrounding context words, while the Skip-gram architecture reverses this task by predicting context words from a target word. Through training on large text corpora, these models learn that words that appear in similar contexts occupy similar positions in the vector space, enabling quantitative measurement of semantic similarity via cosine similarity. CLIP (Contrastive Language–Image Pre-Training; Radford et al., 2021), in contrast, is a multimodal model that learns to align images and natural language descriptions in a shared embedding space. It consists of separate image and text encoders that map inputs into comparable vector representations; the model is trained via contrastive learning to maximize cosine similarity between matching image–text pairs and minimize it for mismatched pairs. This architecture enables direct quantification of visual-semantic coherence—the degree to which a word’s meaning aligns with its visual representation.

These models provide valuable tools for standardizing word choice, especially in morphologically complex languages such as Turkish where traditional frequency-based selection may not adequately account for visual salience and semantic network structure. However, it is not yet possible to show whether word lists generated by computer methods actually improve vocabulary in real classroom settings (Kewenig et al., 2025). Therefore, the pedagogical value of AI-assisted word selection should be tested experimentally.

Another point of debate is whether AI-generated images are as effective as real pictures at teaching vocabulary. Recent studies show that humans struggle to distinguish AI-generated images from real images (Nightingale and Farid, 2022; Roca et al., 2024). This shows that AI-generated images can be used as reliable, consistent visual sources in education. The transition from computational word selection to AI-generated imagery reflects a broader shift in computer-assisted language learning (CALL): from static, pre-prepared materials toward dynamic, AI-mediated content generation that can be adapted to learner needs in real time (Chapelle, 2001; Golonka et al., 2014).

The main perspective of this study is to position AI not only as a tool for material preparation, but as an integral part of a technology-enriched learning ecosystem. The integrated use of Word2Vec and CLIP in the teaching process—from word selection to visual-semantic alignment, scoring, and image generation—transforms technology from a passive preparation tool into an active contributor to language learning (Chapelle, 2001; Golonka et al., 2014). This approach aligns with a fundamental principle in computer-aided language learning (CALL): technology should not only provide static content, but also support learning interactions (Hubbard, 2011).

This study was specifically designed as a quasi-experimental pilot project to test the feasibility of AI-supported vocabulary instruction in an authentic classroom setting. The different target word sets across conditions (words compatible with high CLIP and words compatible with low CLIP) introduce potentially confounding variables that limit causal inference. Although random assignments were used, the word sets varied in tangibility, frequency, or morphological complexity, suggesting that the observed effects may be due to the characteristics of the words themselves rather than the teaching method. Therefore, the current findings should be considered preliminary and confirmed by more controlled studies. These methodological limitations are detailed in the Discussion.

From an educational psychology perspective, understanding how students interact with AI-assisted instruction involves not only learning outcomes but also the motivational, self-regulatory, and cognitive processes that underlie them. The theory of self-determination (Ryan and Deci, 2000) emphasizes how basic psychological needs such as autonomy, competence, and belonging are essential for intrinsic motivation and effective learning. Achievement goal theory (Elliot and McGregor, 2001) distinguishes between mastery-focused goals (aimed at gaining competence) and performance-oriented goals (aimed at demonstrating competence), and describes how students approach challenging tasks. Cognitive load theory (Sweller, 2011), on the other hand, proposes that information presented in different ways can promote learning by reducing unnecessary load when processed correctly. These theories help us understand how AI-based vocabulary education affects not only information transfer but also students’ thoughts, emotions, and self-regulation processes.

Understanding stakeholder perceptions of technology-enabled assessment and training models is critical for sustainable practices. The Technology Acceptance Model (TAM) describes how individuals rate the use of technology based on its perceived usefulness and ease of use (Davis, 1989; Davis and Venkatesh, 2004). Recently, TAM has expanded to explore the adoption of AI-assisted education tools, incorporating concepts such as perceived fairness, trust, and subjective norm (Choung et al., 2024; Zou and Huang, 2023).

This study was conducted using a two-step mixed approach to fill these gaps. The first phase focused on experimentally testing the effectiveness of AI-assisted vocabulary teaching. The second phase assessed participants’ TAM-based perceptions, self-directed learning strategies, performance goals, cognitive load, and underlying psychological needs. This integrated approach offers an opportunity to investigate the psychological mechanisms behind learning outcomes and stakeholder experiences in a comprehensive framework. The findings should be considered preliminary and exploratory given the study’s pilot nature.

## 2 Theoretical framework and literature

### 2.1 Multimodal vocabulary instruction, CALL integration, and the role of AI-generated visuals

Multimodal vocabulary instruction refers to the simultaneous presentation of verbal and visual information to support word learning, grounded in the principle that multiple encoding channels can reinforce memory traces and deepen semantic processing (Paivio, 1986; Kress, 2000). In the context of computer-assisted language learning (CALL), multimodal instruction leverages digital technologies to integrate text, images, audio, and interactive elements, moving beyond static textbook presentations to create dynamic, learner-responsive environments (Chapelle, 2001; Hubbard, 2011). CALL encompasses not only the delivery of language content through technology but also the use of computational tools to analyze learner data, adapt materials, and provide feedback—functions that are increasingly powered by artificial intelligence (Golonka et al., 2014; Li et al., 2025).

The Dual Coding Theory (Paivio, 1986) suggests that the combined use of verbal and visual channels in the processing of concrete words improves long-term learning. In the field of CALL, Al-Seghayer (2001) demonstrated the superiority of dynamic video descriptions over static images, setting an excellent example for teaching multimodal, technology-driven vocabulary. Golonka et al. (2014) examined more than 350 studies on technology-assisted foreign language learning in detail. It was noted that while evidence of effectiveness is limited for many technological tools, computer-aided pronunciation training and conversation-based interactions provide the strongest empirical support. The authors emphasized the importance of using technology, not only for content delivery, but also as an active tool in the learning process.

This work continues the CALL tradition by integrating AI models directly into the educational process. Word2Vec and CLIP do not just create an offline vocabulary; they also provide real-time visual-semantic consistency scores. These scores determine the visual connections that students will encounter, creating a technology-supported learning environment. Through computational semantic analysis, AI actively filters, organizes, and presents vocabulary, providing a more dynamic learning experience than traditional frequency-based offerings.

AI-driven multimodal learning environments support student learning by providing textual, visual, and auditory input simultaneously (Şimşek et al., 2026; Ye and Yan, 2026). According to cognitive load theory (Sweller, 2011), well-designed multimodal instruction reduces unnecessary load by limiting information flow and allocating working memory to basic operations and schema development. On the other hand, poorly organized multimodal presentations—especially for students who are not yet developed—can lead to unnecessary burden and distraction due to the difficulty of efficiently using multiple sources of information.

Distributed semantic models such as Word2Vec, FastText, and GloVe systematically capture semantic similarity by representing words in a continuous vector space (Mikolov et al., 2013; Li et al., 2025). Studies on Turkish show that static incorporation patterns effectively reflect the language’s morphological richness (Sarıtaş et al., 2024). The CLIP (Contrastive Language-Image Pre-Training) model, on the other hand, combines the two approaches in a shared representation space by evaluating the alignment between words and images with cosine similarity (Radford et al., 2021; dos Santos et al., 2023). Kewenig et al. (2025) achieved a high correlation (*r* = 0.93) in automatically estimating the concrete levels of multimodal transformer-based equipment. These findings support the integration of computational measures of concrete into educational decision-making processes.

### 2.2 Vocabulary as a multi-dimensional construct and assessment challenges

Vocabulary is a complex, multi-dimensional construct that extends beyond the fundamental connections between form and meaning. Nation (2001) proposed a comprehensive framework that examines vocabulary across three main dimensions: form (oral, written, and morphological), meaning (form-meaning relationships, networks of concepts, and references), and usage (grammatical functions, coherence, and register constraints). These dimensions include receptive and productive skills; Thus, “knowing a word” is not a simple binary situation, but an interconnected set of advanced skills. This multi-dimensional perspective profoundly influences how vocabulary is evaluated.

The Vocabulary Scale (VKS; Wesche and Paribakht, 1996) represents the “development” approach used in this work, which gradually moves from non-word recognition to efficient use. However, the VKS has received considerable criticism. Bruton (2009) noted that the distinction between “I think I know” (Grade III) and “I know this word” (Grade IV) is not reliably made by students. Waring (1999) argues that self-reporting tools such as VKS can conflate recognition with recall; he notes that students may get the impression that they know a word only partially before reaching full productivity levels. Schouten-van Parreren (1996), on the other hand, argues that vocabulary is highly contextual; he stresses that tests without context may not capture the full depth of students’ mental vocabulary.

Several alternative evaluation methods have been developed to eliminate these limitations. Read's (2000) Word Associates Form (WAF) uses network information and measures the depth of knowledge by enabling learners to identify semantic and transversal relationships; Here, not only form-meaning relationships, but also relational structures emerge. Laufer and Nation's (1999) Generative Word Level Test (PVLT) measures productive capacity through retrieval tasks, providing a more detailed measure of productivity than VKS. These reviews suggest that while VKS retains its practical value in a classroom, it can remain superficial in vocabulary and needs to be supplemented with more precise tools in research settings.

### 2.3 Self-regulated learning, achievement goals, and the MSLQ framework

Self-directed learning (SRL) reflects how actively students engage in their learning at the metacognitive, motivational, and behavioral levels (Zimmerman, 2002). The Motivated Strategies for Learning Questionnaire (MSLQ; Pintrich, 2004; Pintrich et al., 1991) provides a broad framework encompassing the two basic elements of SRL: motivation and learning strategies.

The motivation section of the MSLQ consists of three basic subscales. It consists of a total of six subscales: (a) value components (intrinsic goal value, external goal orientation, and task value), (b) expectation components (management beliefs and self-efficacy), and (c) emotional components (test anxiety). The learning strategy section consists of two main sections with nine subscales: (a) cognitive and metacognitive strategies (repetition, explanation, organization, critical thinking, and metacognitive self-regulation) and (b) resource management strategies (time and work environment management, effort regulation, peer learning, and seeking help).

Research shows that self-managed students who use deep-processing strategies (explanation, organization, critical thinking) and effectively manage learning resources achieve better academic outcomes than students who rely on superficial strategies (e.g., iteration) (Credé and Phillips, 2011). In the context of AI-assisted vocabulary education, SRL is particularly important because technology offers several benefits by providing structured support to learners with different SRL profiles. Students with high self-efficacy and strong metacognitive self-regulation can benefit more from the visual-semantic configurations provided by CLIP-generated word-visual pairs; students with low SRL levels may need additional support.

Performance goal theory is a dynamic framework that explains how goals related to students’ perceptions of competence affect their cognitive, emotional, and behavioral engagement in learning tasks (Elliot and McGregor, 2001). The 2 × 2 performance goal model distinguishes four different types of goals based on the competence (control and performance) definition and the competence value (approach and avoidance):

- *Goals for a mastery approach*: Focus on improving competence, undertaking tasks, and promoting self-improvement. Deep learning is associated with continuous improvement and a constructive response to challenges.
- *Mastery avoidance goals*: Focus on avoiding misunderstandings or completing the task completely. Although it is less recommended, it can be especially useful for perfectionist students.
- *Performance-based goals*: Focus on comparing yourself to others and creating positive expectations. It can stimulate effort and success, but it can also increase anxiety and lead to superficial functioning.
- *Performance avoidance goals*: Focus on avoiding poor performance compared to others. Anxiety is associated with negative consequences such as disconnecting and avoiding help.

In the context of AI-driven vocabulary learning, learners with mastery- and performance-focused goals may differ in their approaches. Mastery-focused learners may see AI-selected words as an opportunity for systematic skill development; Performance-focused students may try to outperform others in vocabulary tests. Understanding these different goal orientations can help inform the design of AI-driven learning environments that promote adaptive goal adoption.

### 2.4 Cognitive load theory, self-determination theory, and technology acceptance

Cognitive Load Theory (CLT; Sweller, 2011) highlights the limited capacity of working memory and emphasizes the importance of instructional designs to distribute cognitive resources across the three types of load optimally:

- *Innate cognitive load*: Reflects the natural complexity of the learning material and the student’s prior knowledge. For example, acquiring new words while learning a new language requires a high internal load.
- *Unnecessary cognitive load*: Associated with poorly designed teaching materials that are not focused on learning. Confusing or unnecessary presentations can add to this type of stress.
- *Germane cognitive load*: Focuses on creating and automating schemas; refers to productive processing that supports learning.

By presenting information in both visual and verbal forms (modal effect), a well-designed multimodal presentation can reduce unnecessary stress and respect the different abilities of working memory (Paivio, 1986). However, if the same information is presented in different methods, the contingency effect can kick in and increase overheads. The specialization reversal effect (Kalyuga, 2011) indicates that teaching methods that are useful to beginners can be unnecessary or even detrimental to more experienced students.

Through AI, CLIP provides highly coherent word-visual connections that support meaning construction and reinforcement of meaning structure. This eliminates the need for students to search for mental visual resources while easing unnecessary burdens. The mental effort scale developed by Paas (1992) provides a validated, one-dimensional measure of mental investment in light of task complexity and variations in teaching design.

The theory of self-determination (SDT; Ryan and Deci, 2000) emphasizes that it is essential to meet three basic psychological needs in order to be optimally motivated and stay in a good mood: autonomy (acting of one’s own will and self-affirmation), competence (sense of achievement and competence), and connectedness (bonding and relationship development). When these needs are met, individuals strengthen their innate motivation; They participate in activities they love or appreciate. When not met, situations such as controlled motivation or lack of motivation can arise, driven by external pressures or guilt.

In educational settings, supportive environments provide meaningful options, appropriate difficulty levels, and warm interpersonal interactions; environments that block needs can be controlling or overwhelming, or can lead to social isolation. Learning environments powered by AI technology provide autonomy by offering personalized word choices and self-directed learning opportunities; increase the sense of competence through direct feedback and structured challenges; and foster cooperation. Social interactions through teachers can strengthen the relationship. However, excessive algorithmic control or cold feedback can reduce feelings of autonomy and belonging; This highlights the importance of maintaining a balance between technological efficiency and human relationships.

The Technology Acceptance Model (TAM) is one of the most widely used theoretical frameworks for understanding how users adopt new technologies (Davis, 1989). The perceived usefulness and ease of use in the model influence consumer attitudes and behavioral intentions towards the technology. Venkatesh and Davis (2000) extended the model by adding cognitive constructs, such as subjective norm. Recent research shows that TAM is also being used successfully in the field of artificial intelligence; It shows that perceived usefulness and ease of use significantly predict attitudes and intentions (Sohn and Kwon, 2020; Zou and Huang, 2023).

In the context of assessment, the relationship between perceived equity, motivation, and learning outcomes is crucial (Colquitt et al., 2001). In a meta-analysis by Gavrilau et al. (2025) on the effectiveness of online testing in language acquisition, it was found that students’ attitudes towards online testing vary depending on the type of application and context. McKinley and Rose (2026) found that cultural differences significantly affect the effectiveness of AI-based feedback systems in multicultural settings. Rossic (2026) noted that while English teachers generally approach AI-driven assessment tools positively, they express concerns and cautious attitudes towards educational management. Li (2025) found that while automated writing grading systems can alleviate teachers’ workload, teachers have mixed feelings towards these systems due to time-saving and quality issues.

This study considers TAM in the context of language assessment and emphasizes perceived equality as a predictor. Theoretically, a grading format that does not seem fair, no matter how technically advanced, may not provide the necessary motivation to learn.

### 2.5 Synthesis: theoretical gaps and the present study

The theoretical frameworks reviewed above—multimodal vocabulary instruction, CALL integration, self-regulated learning, achievement goal theory, cognitive load theory, self-determination theory, and TAM—converge on a central question: How does AI-mediated visual-semantic coherence shape vocabulary learning outcomes and stakeholder experiences? Despite substantial theoretical development, empirical research examining the integration of computational word selection (via Word2Vec and CLIP) with educational psychology constructs in authentic classroom settings remains scarce. Specifically, three gaps motivate the present study: (a) the lack of classroom-based empirical testing of AI-selected word lists against traditional frequency-based approaches; (b) unresolved questions about the equivalence of AI-generated and authentic visual materials in language learning; and (c) limited understanding of the motivational, self-regulatory, and cognitive mechanisms that mediate AI-assisted vocabulary learning. This pilot study addresses these gaps through a two-phase mixed-methods design that combines quasi-experimental learning outcomes with comprehensive psychological assessments.

**Figure 1:**
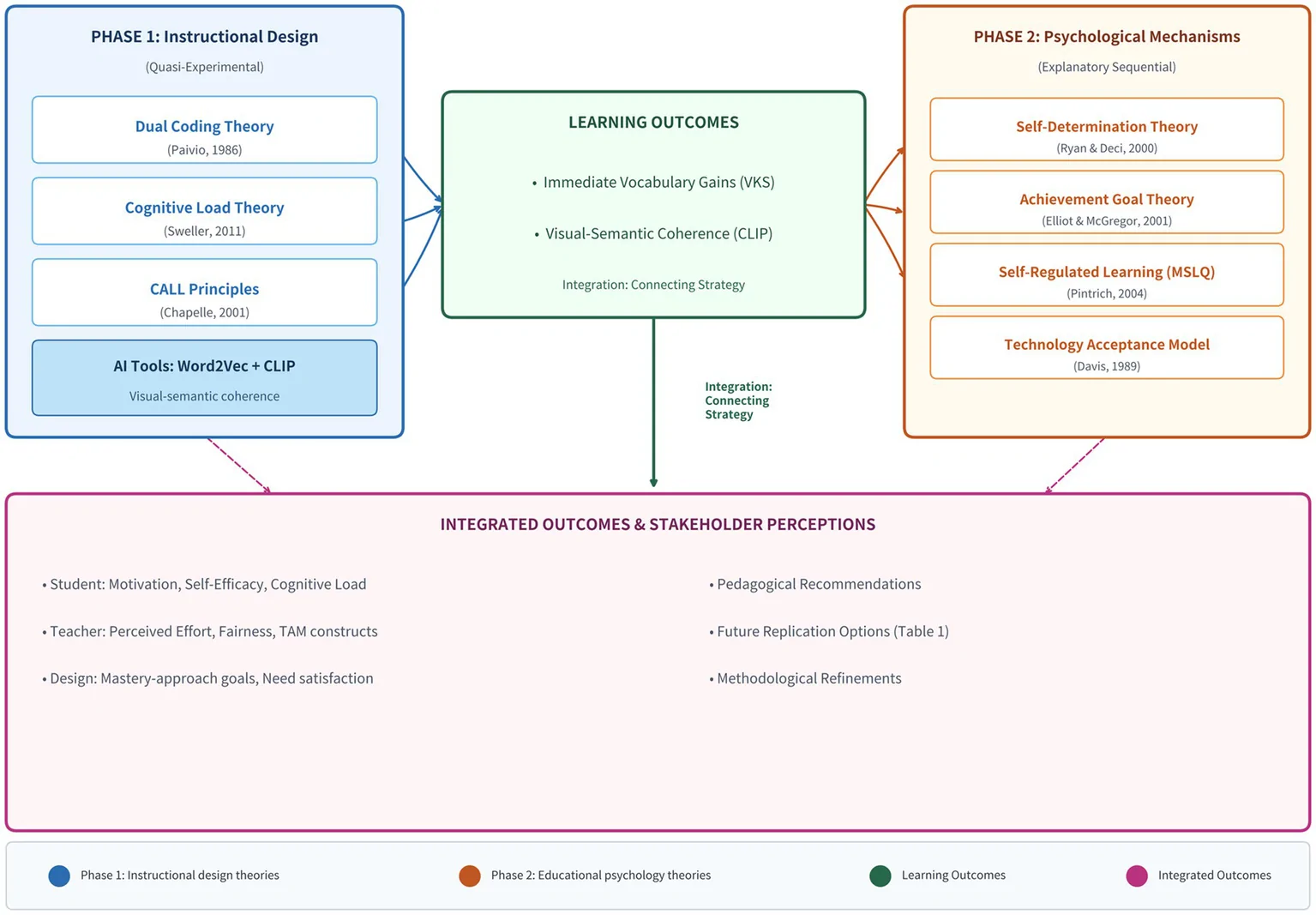
Conceptual Framework: Theoretical Interactions in AI-Supported Vocabulary Instruction. Source: Adapted by the authors from Paivio (1986), Sweller (2011), Chapelle (2001), Ryan and Deci (2000), Elliot and McGregor (2001), Pintrich (2004), Davis (1989), and Fetters et al. (2013). The figure represents an original synthesis and adaptation of the theoretical concepts presented in these sources and was not reproduced from any single previously published figure.

Figure 1 illustrates the integrated conceptual framework guiding this study. Phase 1 (Instructional Design) is informed by Dual Coding Theory, Cognitive Load Theory, and CALL principles, which guide the development of AI-mediated materials using Word2Vec and CLIP. Phase 2 (Psychological Mechanisms) draws upon Self-Determination Theory, Achievement Goal Theory, Self-Regulated Learning (MSLQ), and the Technology Acceptance Model to explain stakeholder experiences. Learning Outcomes serve as the bridge between instructional design and psychological mechanisms. Integrated Outcomes emerge from the joint interpretation of both phases, yielding pedagogical recommendations, options for future replication (Table 1), and methodological refinements. The thick arrow connecting Learning Outcomes to Integrated Outcomes represents the connecting strategy (Fetters et al., 2013), whereby Phase 1 outcomes purposively guide Phase 2 instrument selection and interpretation. Solid arrows indicate direct theoretical influence; dashed arrows indicate contributions to integrated recommendations.

**Table 1:** Design options for future replication.

Option	Description
A (Ideal)	All groups learn identical 50-word sets; manipulation limited to visual condition (AI image, real photo, textbook) and concordance level—2 × 3 factorial design.
B (Practical)	If different words must be used, match on psycholinguistic variables (frequency, length, concreteness, morphological complexity) and control via ANCOVA.

## 3 Method

### 3.1 Research design

This study was conducted in two phases using an explanatory sequential mixed approach (Creswell and Plano Clark, 2018). In the first phase, a quasi-experimental design with three conditions (pre-test/post-test, two experimental groups, and a control group) was used to evaluate the effectiveness of AI-assisted instruction in concrete vocabulary. The three conditions are: (a) words with high CLIP scores + AI images (AI-Image group), (b) words with high CLIP scores + real photos (Real-Photo group), and (c) words with low CLIP scores + traditional textbook images (control group). This design is quasi-experimental because different target word sets are used between the terms, creating a potentially confounding variable that limits causal inferences (Table 1).

The explanatory sequential design uses Phase 1 results to purposively guide Phase 2 instrument selection and interpretation. Phase 2 was designed to explain and contextualize Phase 1 results by identifying the psychological mechanisms underlying observed learning outcomes, thereby achieving integration through the connecting strategy (Fetters et al., 2013).

In the second stage, a comprehensive quantitative survey was conducted to examine participants’ perceptions of the technology-assisted assessment and instruction model, incorporating constructs from TAM, self-regulated learning (MSLQ), achievement goals (AGQ), cognitive load (Paas scale), and basic psychological needs (SDT).

### 3.2 Participants

In the first phase of the study, 60 international students at the A1–A2 level in the Turkish Preparatory Unit at a university in Turkey participated. Participants were deliberately selected and randomly assigned to three conditions. The AI-Image group showed no significant differences in age and prior Turkish knowledge (*n* = 15; 8 females, 7 males; M = 21.3, SD = 2.2), the Real-Photo group (*n* = 15; 8 females, 7 males; M = 21.5, SD = 1.8) and the control group (*n* = 30; 15 females, 15 males; M = 21.7, SD = 1.9) (*p* > 0.05). The control group was selected larger (*n* = 30) in an attempt to increase the statistical power of the baseline comparison between computational and traditional word choice. Two experimental subgroups (*n* = 15 each) provided sufficient power for image type comparison by prior power analysis (G*Power; *f* = 0.40, *α* = 0.05, power = 0.80). However, with n = 15 per group, the design was directed to detect only large effects (d ≥ 0.75); Smaller but educationally significant differences between AI and real photo conditions can be overlooked.

Randomization was performed by an independent research assistant who was not involved in instruction, using a computer-generated random sequence (random.org) stratified by prior Turkish proficiency (pre-test VKS scores) to ensure balanced groups. The randomization procedure was conducted after baseline assessment but before the intervention began.

Instructors (*n* = 3) were full-time Turkish language teachers with an average of 5.3 years of teaching experience (SD = 2.1). Two instructors held an MA in Applied Linguistics, and one held an MA in Educational Technology. All instructors had previously used the university’s learning management system and had received a 2-h training session on the AI-supported platform prior to the intervention. Instructors were randomly assigned to experimental conditions using the same stratified randomization procedure applied to students.

In the second phase, in addition to the 60 students from the first phase, 15 Turkish teachers working at the same institution with at least 2 years of experience participated (9 female, 6 male; M = 38.2, SD = 6.4). All teachers had experience in traditional, alternative, and online grading models.

### 3.3 Ethical considerations

This study was approved by the Ethics Committee of Girne American University (Protocol No: 2025-26/035; Date: March 3, 2026). All participants submitted written informed consent prior to data collection. Students were informed that participation was voluntary and would not affect their grades. The data were anonymized and stored securely in accordance with the General Data Protection Regulation (GDPR).

### 3.4 Intervention and materials

#### 3.4.1 Intervention protocol

The intervention consisted of 4 weeks of structured vocabulary instruction delivered through a web-based learning platform. Students in all three conditions received two 45-min sessions per week, for a total of eight sessions. Each session followed a standardized structure: (a) 10 min of pre-session review using spaced repetition of previously encountered words; (b) 25 min of new word presentation, during which target words were introduced with their visual referents and semantic neighbors; (c) 5 min of interactive matching exercises where students paired words with images; and (d) 5 min of formative feedback summarizing correct responses and highlighting errors. The platform automatically logged student interactions, including time on task, response accuracy, and number of repetitions requested.

Teachers served as facilitators rather than primary instructors during the intervention. Their role involved: (a) introducing the platform and explaining navigation procedures in the first session; (b) monitoring student engagement and providing technical assistance as needed; (c) leading brief whole-class discussions at the end of each session to address common difficulties; and (d) ensuring adherence to the protocol across all sessions. Teachers did not select target words or generate visual materials; these were pre-programmed by the research team to standardize content delivery across conditions.

#### 3.4.2 Phase 1: vocabulary tests and visual material

*Pretest and posttest*: A 50-item “Vocabulary Scale” (VKS; Wesche and Paribakht, 1996; adapted to Turkish) was used to evaluate participants’ knowledge of the target words in terms of meaning, usage, and efficiency. The scale uses a 5-point system (1 = I have never seen the word, 5 = I can use the word correctly in a sentence). The VKS is primarily a self-report tool that assesses learners’ familiarity with the connections between form and meaning, rather than their deep, meaningful knowledge of word use in context (Bruton, 2009; Waring, 1999). The scale was applied in the pre-testing phase before the intervention. Then, after 4 weeks of training, it was administered again. The results were obtained from post-delayed testing and therefore reflected immediate gains in learning. Additional measures proposed for future studies include the Recall/Recognition Test, a 50-item multiple-choice format that evaluates knowledge of format meaning. The Controlled Production Test was adapted from Laufer and Nation (1999), and the first letters were presented in sentence contexts.

*Visual materials*: In the experimental groups, a total of 25 concrete names were selected, each with a visual semantic compatibility score of 0.90 or higher from the CLIP model. These names include common things in our daily lives, such as tomatoes, butter, trains, teachers, and books. In the AI-Image group, each word was paired with AI-generated images (produced using Midjourney v6). In the Real-Photo group, each word was associated with a real photo (via the Google Custom Search API). The control group was shown 25 random words with a CLIP score below 0.70 and traditional textbook images. All three groups used the same web-based vocabulary learning platform; thus, the technology-based learning environment has always remained the same. Only the content (word choice and image source) has changed. This use of different word sets means that words (with high or low CLIP scores) can reflect characteristics such as natural concreteness, frequency, and semantic clarity; This is not a computational selection method.

During the word selection process, the 50 most common names at the CEFR A1-A2 level were selected using the Turkish Foreign Language Corpus (TaSL) on the TS Corpus platform, and the ones that were close in meaning were defined by the Word2Vec (CBOW and Skip-gram) method. The target words, frequencies, semantic neighbors, and distribution matching ratios obtained in this process are shown in Table 2.

**Table 2:** Target words, frequencies and semantic neighbors (Word2Vec).

Target word	Frequency	W2V neighbor	CBOW relevance rate	Skipgram relevance rate
doktor (doctor)	71	hasta (patient)	0.61	0.68
öğrenci (student)	60	öğretmen (teacher)	0.71	0.70
kitap (book)	58	gazete (newspaper)	0.62	0.68
ders (lesson)	51	sınav (exam)	0.62	0.59
çay (tea)	40	süt (milk)	0.65	0.73
araba (car)	28	motosiklet (motorcycle)	0.50	0.58
deniz (sea)	26	göl (lake)	0.55	0.66
otobüs (bus)	24	taksi (taxi)	0.70	0.80
domates (tomato)	23	salatalık (cucumber)	0.77	0.85
el (hand)	22	kol (arm)	0.56	0.64
yağmur (rain)	22	kar (snow)	0.60	0.70
tavuk (chicken)	22	kabak (pumpkin)	0.70	0.66
ekmek (bread)	21	peynir (cheese)	0.67	0.69
yumurta (egg)	19	tereyağı (butter)	0.60	0.77
çiçek (flower)	18	ağaç (tree)	0.52	0.58
sebze (vegetable)	18	meyve (fruit)	0.50	0.70
eczane (pharmacy)	17	hastane (hospital)	0.65	0.63
polis (police)	17	şoför (driver)	0.52	0.64
sınıf (classroom)	17	öğrenci (student)	0.51	0.58
bulut (cloud)	17	güneş (sun)	0.68	0.69
tiyatro (theater)	16	sinema (cinema)	0.60	0.77
ayran (ayran)	14	köfte (meatball)	0.56	0.78
elma (apple)	14	patlıcan (eggplant)	0.72	0.75
eşya (furniture/item)	14	mobilya (furniture)	0.56	0.64
kuş (bird)	14	at (horse)	0.53	0.56
peynir (cheese)	14	yumurta (egg)	0.70	0.78
kış (winter)	13	sonbahar (autumn)	0.65	0.72
uçak (airplane)	13	otobüs (bus)	0.63	0.69
gemi (ship)	12	tren (train)	0.53	0.63
mercimek (lentil)	12	fasulye (bean)	0.69	0.81
tabak (plate)	12	karabiber (black pepper)	0.71	0.69
masa (table)	11	dolap (cabinet)	0.57	0.69
otel (hotel)	11	havaalanı (airport)	0.53	0.50
yoğurt (yogurt)	11	makarna (pasta)	0.61	0.71
banyo (bathroom)	10	mutfak (kitchen)	0.53	0.73
tereyağı (butter)	10	peynir (cheese)	0.63	0.80
göl (lake)	9	dağ (mountain)	0.52	0.55
koyun (sheep)	9	tavşan (rabbit)	0.56	0.58
kayak (ski)	9	tenis (tennis)	0.55	0.66
köy (village)	9	düğün (wedding)	0.52	0.53
rüzgar (wind)	9	yağmur (rain)	0.60	0.60
soğan (onion)	9	patates (potato)	0.70	0.85
kapı (door)	8	pencere (window)	0.54	0.67
koltuk (sofa/armchair)	8	sandalye (chair)	0.55	0.58
saç (hair)	8	gözlük (glasses)	0.57	0.58
tren (train)	8	tramvay (tram)	0.71	0.76
yüz (face)	8	göz (eye)	0.59	0.58
madalya (medal)	8	olimpiyat (olympics)	0.52	0.55
saray (palace)	7	cami (mosque)	0.61	0.71
baş (head)	7	ağız (mouth)	0.51	0.68

In Table 2, the Turkish word is given first in each line, followed by its English equivalent in parentheses. The CBOW and Skip-gram similarity rates reflect the cosine similarity in the distributed vector space trained with the Word2Vec model on the TS Corpus platform. Complete frequency distributions are available in the supplementary materials.

The technical background of the computational selection process can be summarized as follows: The semantic neighbors of the target names were determined using the Word2Vec model (300-dimensional vectors, 5-word context window, CBOW and Skip-gram architectures, negative sampling) trained in the TaSL corpus on the TS Corpus platform (14 texts, 121,411 tokens, 22,600 unique entries). OpenAI’s CLIP (ViT-B/32, 512-dimensional common space) model was used to evaluate visual semantic congruence. Words calculated on 224 × 224-pixel images and scoring above the 0.90 threshold were selected for the experiment. The CLIP scoring engine was integrated into the learning platform; thus, students in the experimental groups received real-time visual-semantic compatibility feedback during vocabulary exercises, while the control group did not receive such computational support. All analyses were performed with Python 3.9, transformers 4.25.1, and PyTorch 1.13.0.

#### 3.4.3 Phase 2: Educational Psychology questionnaires

In the second phase, a validated series of questionnaires was used to assess multiple dimensions of the learning experience. All items were rated on a 5-point Likert scale (1 = Strongly Disagree, 5 = Strongly Agree), unless otherwise noted.

a. *TAM-based observation scale.* Based on Davis (1989) and Davis and Venkatesh (2004), this scale consisted of five dimensions: (1) motivation (5 items), (2) perceived fairness (5 items), (3) effort required (5 items, reverse-coded), (4) perceived usefulness (5 items), and (5) perceived ease of use (5 items). Internal consistency coefficient (Cronbach’s alpha) was 0.94.

*Sample items*:

1. “The AI-powered vocabulary encourages me to take a closer interest in the lesson.” (Motivation)
2. “AI-assisted assessment ensures a fair process for all students.” (Perceived justice)
3. “I have to spend a lot of time on AI-powered vocabulary training.” (Effort required; reverse-coded)

b. *Motivated Learning Survey Strategies (MSLQ).* MSLQ (Pintrich, 2004; Pintrich et al., 1991) has been adapted to assess self-regulated learning in this context. Given the pilot nature of this study, an abbreviated version of 44 items was used, which stipulated that:

*Motivation scales:*

1. Intrinsic Goal Orientation (4 items): “I prefer course material that is really challenging to learn new things.”
2. Extrinsic Goal Orientation (4 items): “Getting a good grade in this class is the most satisfying thing I have right now.”
3. Task Value (4 items): “I think I can use what I learned in this course in other lessons.”
4. Control beliefs (4 items): “If I study in appropriate ways, I can learn the material.”
5. Self-efficacy (4 items): “I believe I will get a perfect grade in this class.”
6. Exam anxiety (4 items): “I feel uncomfortable and sad while taking the exam.”

*Learning strategies scales:*

1. Use of cognitive strategies (repetition, explanation, organization; 12 items)
2. Self-regulation (metacognitive self-regulation, time/work environment, exercise regulation; 12 items)

c. *Performance Target List (AGQ).* AGQ (Elliot and McGregor, 2001) was adapted to evaluate performance goals in the context of AI-mediated word learning:
  1. Mastery approach (3 items): “I want to learn as much as I can from this vocabulary course.”
  2. Avoiding Mastery (3 items): “I am worried that I will not learn everything I can in this course.”
  3. Performance approach (3 items): “For me, it is important to perform better than other students.”
  4. Avoiding performance (3 items): “My goal is to avoid performing worse than others.”
d. *Cognitive Load Scale.* 9-point mental effort scale (Paas, 1992; Paas et al., 2003) was applied after each learning session by assessing mental effort: “How much mental effort did you put into the vocabulary learning task?” (1 = Very, very low mental effort, 9 = Very, very high mental effort). In addition, the subjective cognitive load scale consisting of three items was evaluated:
  1. Internal blame: “The vocabulary was complex and difficult to understand.”
  2. Unnecessary accusation: “The way the vocabulary was presented was confusing.”
  3. Germane load: “The visual material helped me understand the words better.”
e. *Basic Psychological Needs Scale (BPNS).* Adapted from the work of Ryan and Deci (2000), this scale assessed the need for satisfaction in the context of learning:
  1. Autonomy (4 items): “I felt like I could make my own choices about how to study vocabulary.”
  2. Proficiency (4 items): “I felt competent and effective in learning the vocabulary of words.”
  3. Consistency (4 items): “I bonded with my teacher and classmates during vocabulary lessons.”

### 3.5 Data analysis

In the first stage, the data were analyzed with SPSS 30.0. A one-way ANOVA was used to test differences among the three cases, followed by Tukey’s *post hoc* HSD tests for pairwise comparisons. A paired-samples *t*-test was used to examine differences between pre- and post-tests within each group. The effect sizes were calculated using Cohen’s d for the paired t-tests and the partial ETA square (ηp^2^) for ANOVA.

In the second phase, descriptive statistics, independent sampling t-tests (student-teacher comparisons), Pearson correlation coefficients, and hierarchical multiple regression analyses were performed. Regression models examined whether performance goals and basic psychological needs predicted vocabulary acquisition and motivation, with previous Turkish knowledge taken into account. The significance level for all analyses was determined as *p* < 0.05.

*Retention Rate Formula for Future Studies:* For repeat studies with delayed tests, the retention rate should be calculated as follows:

$$\begin{array}{c} \text{Retention rate}=(\text{After delayed test}-\mathrm{Pre}-\text{test})\\ /(\text{Immediately after test}-\mathrm{Pre}-\text{test})\times 100 \end{array}$$

## 4 Results

### 4.1 Phase 1: results of the experimental training study

#### 4.1.1 Pretest–posttest comparisons

One-way ANOVA in post-test scores showed that the group main effect was significant: *F*(2, 57) = 78.42, *p* < 0.001, *ηp^2^* = 0.73. Tukey’s post-HSD tests showed that both the AI-Image group (*M* = 39.13, *SD* = 4.71) and the Real-Photo group (*M* = 38.33, *SD* = 4.42) performed significantly better than the control group (*M* = 24.60, *SD* = 3.89), *p* < 0.001. The effect size of the combined experimental groups relative to the control group was *d* = 1.42 (95% CI [1.02, 1.82]), indicating a large but plausible effect consistent with meta-analytic findings in multimedia word CALL research (Al-Seghayer, 2001; Golonka et al., 2014). There was no significant difference between the AI-Image and Real-Photo groups (*p* = 0.678). However, given the small sample size (*n* = 15 per group), this null finding may reflect insufficient statistical power rather than true equivalence; the design was powered to detect only large effects (d ≥ 0.75), and smaller but educationally meaningful differences cannot be excluded. The confusion of word sets (high CLIP vs. low CLIP) suggests that these effects may be attributable to inherent differences between word pools rather than to instruction manipulation alone (see Table 3).

**Table 3:** Pre- and post-test scores of all groups.

Group	Test	*n*	*M*	*SD*	*t*	*p*
AI image	Pre-test	15	12.33	3.15	−16.84	<0.001
	After the exam	15	39.13	4.71		
Real photo	Pre-test	15	12.47	3.28	−15.92	<0.001
	After the exam	15	38.33	4.42		
Control	Pre-test	30	12.87	3.45	−9.87	<0.001
	After the exam	30	24.60	3.89		

These results only reflect direct learning outcomes. Evaluation following a delayed test cannot be considered long-term.

#### 4.1.2 Comparing AI images and real photos

As noted above, there was no significant difference in post-test scores between the AI-Image group (*n* = 15) and the Real-Photo group (*n* = 15); *t*(28) = 0.42, *p* = 0.678. The AI image group scored *M* = 39.13 (*SD* = 4.71); the real photo group scored *M* = 38.33 (*SD* = 4.42). However, teachers’ observations and students’ feedback showed that AI-generated images provided a more standardized presentation in terms of color consistency, background control, and representation of cultural objects. This finding suggests that AI images could offer a practical solution to the variability problem of web-based photos. This non-significant difference should not be interpreted as evidence of equivalence; rather, it indicates that any true difference, if present, is smaller than the study was designed to detect.

#### 4.1.3 Results of the computational selection process

Computational analyses showed that the mean CLIP confidence score for 50 target names and 500 semantic neighbors was 0.89 (*SD* = 0.04, range 0.82–0.96); 73% of neighbors scored above 0.90. Food and beverage terms had the highest mean (*M* = 0.95; e.g., tomato–cucumber: 0.97; egg–butter: 0.96), while transport words (*M* = 0.94) and occupation names showed strong visual-semantic concordance. High consistency (*r* = 0.78, *p* < 0.001) was observed between the CBOW and Skip-gram architectures; Skip-gram captured more concrete neighbors for polysemic elements. Visual coherence was high in concrete nouns (*SD* < 0.05), while more variation was found in culturally variable terms (*SD* > 0.10).

### 4.2 Phase 2: results of Educational Psychology

#### 4.2.1 TAM-based perception results

According to Table 4, students perceived the AI-assisted model as moderately motivating (*M* = 3.52), whereas teachers reported lower motivation (*M* = 2.89); this difference was significant: *t*(73) = 2.89, *p* = 0.005. Both groups rated the model as reasonable; No significant difference was found between the groups (*p* = 0.464). On the effort dimension, teachers (*M* = 3.67) saw the model as more demanding than students (*M* = 2.94), *t*(73) = −3.12, *p* = 0.003.

**Table 4:** Student and teacher perception scores (TAM dimensions).

Dimension	Group	*n*	*M*	*SD*	*t*	*p*
Motivation	Student	60	3.52	0.74	2.89	0.005
	Teacher	15	2.89	0.81		
Perceived honesty	Student	60	3.61	0.68	0.74	0.464
	Teacher	15	3.48	0.72		
Effort required	Student	60	2.94	0.79	−3.12	0.003
	Teacher	15	3.67	0.85		
Usability	Student	60	3.78	0.71	1.15	0.254
	Teacher	15	3.52	0.76		
Ease of use	Student	60	3.45	0.82	0.62	0.540
	Teacher	15	3.33	0.69		

#### 4.2.2 Self-regulated learning (MSLQ) outcomes

The experimental groups reported significantly higher rates of intrinsic goal orientation, task value, self-efficacy, use of cognitive strategies, and self-regulation compared to the control group. These associations are correlational; whether the instructional manipulation caused these differences cannot be determined without pretest MSLQ data. Interestingly, intrinsic goal orientation was positively correlated with vocabulary acquisition (*r* = 0.48, *p* < 0.01), indicating that students who naturally found the material interesting achieved better learning outcomes (see Table 5).

**Table 5:** MSLQ subscale scores by group.

MSLQ subscale	AI Image	Real-Photo	Control	*F*	*p*
Intrinsic goal orientation	3.89 (0.72)	3.76 (0.68)	3.21 (0.81)	6.42	0.003
Extrinsic target orientation	3.45 (0.85)	3.52 (0.79)	3.67 (0.74)	0.58	0.564
Job value	4.12 (0.65)	4.08 (0.71)	3.45 (0.82)	7.89	0.001
Control beliefs	3.78 (0.69)	3.71 (0.74)	3.34 (0.78)	2.84	0.067
Self-efficacy	3.56 (0.81)	3.48 (0.76)	2.98 (0.89)	3.52	0.036
Test anxiety	2.34 (0.92)	2.41 (0.88)	2.89 (0.95)	2.61	0.082
Use of cognitive strategies	3.67 (0.74)	3.58 (0.69)	3.12 (0.81)	4.12	0.021
Self-regulation	3.45 (0.78)	3.38 (0.72)	2.87 (0.85)	3.89	0.027

These group differences may reflect preexisting differences in self-regulated learning tendencies rather than effects of the instructional manipulation, as the MSLQ was administered only after the intervention. Causal attribution to the experimental conditions is therefore not warranted.

#### 4.2.3 The results of performance target (AGQ)

Mastery approach objectives showed the strongest positive association with vocabulary acquisition, consistent with theoretical predictions that learning-focused goals promote deep processing and persistent engagement. Performance-avoidance targets were negatively associated with gains, suggesting that fear of poor performance can undermine word acquisition in AI-mediated contexts. These correlations do not establish causality; they identify patterns that warrant experimental investigation in future research (see Table 6).

**Table 6:** Performance target correlations with vocabulary gain.

Performance target	*r* with words coincidence	*p*
Mastery approach	0.48	<0.001
Control-avoidance	0.12	0.342
Performance approach	0.31	0.016
Performance avoidance	−0.24	0.048

#### 4.2.4 The results of cognitive load

The experimental groups reported significantly less mental effort and outward loading, and significantly higher germane load compared to the control group. This pattern suggests that the highly coherent word-image connections selected by CLIP may reduce unnecessary cognitive load and free up working memory for efficient schema construction (germane load) by providing semantically aligned visuoskeletal cues. However, given the quasi-experimental design and post-hoc measurement, this interpretation is speculative and requires confirmation through controlled experimental studies (see Table 7).

**Table 7:** Cognitive load by group.

Cognitive load dimension	AI image	Real-photo	Control	*F*	*p*
Mental Effort (Easter)	4.2 (1.8)	4.5 (1.9)	5.8 (2.1)	4.89	0.011
Intrinsic load	3.8 (0.9)	3.9 (0.8)	4.5 (1.0)	3.76	0.029
Unnecessary burden	2.1 (0.8)	2.3 (0.9)	3.2 (1.1)	8.92	<0.001
Germane Load	4.5 (0.9)	4.3 (1.0)	3.1 (1.2)	12.34	<0.001

#### 4.2.5 Results of the basic psychological needs (SDT)

Hierarchical multiple regression analysis showed that basic psychological needs significantly predicted intrinsic motivation: *R^2^* = 0.52, *F*(3, 56) = 20.34, *p* < 0.001. Competence emerged as the strongest predictor (*β* = 0.62, *p* < 0.001), followed by autonomy (*β* = 0.31, *p* = 0.012). Relatedness did not contribute uniquely (*β* = 0.14, *p* = 0.187). This pattern supports SDT predictions, with competency satisfaction being particularly critical for motivation in structured learning tasks. These regression results identify statistical associations, not causal effects; the directionality of relationships between needs and motivation cannot be established with cross-sectional data (see Table 8).

**Table 8:** Basic psychological needs and motivation.

Variable	*M*	*SD*	*r* with intrinsic motivation	*p*
Autonomy	3.67	0.82	0.45	<0.001
Competence	3.89	0.74	0.58	<0.001
Relationship	3.45	0.91	0.38	0.003

#### 4.2.6 Correlation analysis

Correlation analyses showed a strong positive association between motivation and effort (*r* = 0.51, *p* < 0.01) and a moderately positive association between motivation and perceived honesty (*r* = 0.42, *p* < 0.01). Furthermore, a strong association was found between perceived fairness and usefulness (*r* = 0.55, *p* < 0.01). Interestingly, competency satisfaction (SDT) showed the strongest correlation with motivation (*r* = 0.71), highlighting the central role of perceived effectiveness in AI-mediated learning contexts. These correlations are descriptive associations that do not imply causal direction or experimental manipulation effects (see Table 9).

**Table 9:** Summary of key correlations (TAM, MSLQ, AGQ, SDT).

Variable	1	2	3	4	5
1. Motivation (TAM)	—				
2. Perceived fairness	0.42**	—			
3. Self-efficacy (MSLQ)	0.62**	0.48**	—		
4. Mastery approach (AGQ)	0.55**	0.41**	0.67**	—	
5. Competence (SDT)	0.71**	0.52**	0.78**	0.72**	—

*p < 0.05, **p < 0.01.

## 5 Discussion

This two-phase pilot study aimed to empirically test the pedagogical effectiveness of AI-assisted multimodal education in teaching concrete vocabulary and to examine the educational psychology mechanisms behind stakeholder experiences. The findings provide important clues regarding both learning outcomes and the motivational, self-regulatory, and cognitive processes that mediate them; however, all results should be considered preliminary due to the quasi-experimental design and the absence of delayed testing.

### 5.1 The effectiveness of AI-powered vocabulary teaching

The findings of phase one showed that presenting concrete words with high visual-semantic coherence, as determined by the CLIP and Word2Vec models, with visual support was significantly more effective than traditional/random word lists. Still, the use of different word sets between conditions is the most critical limitation of this study. Since different target words were used in the experimental (high CLIP) and control (low CLIP) conditions, the observed effects may reflect differences in word properties such as natural tangibility, imaginability, or frequency, rather than differences in the computational selection method. Future studies should either use the same word sets with different presentation methods (Option A) or match and control psycholinguistic variables (Option B).

The three-condition design suggests that we might attribute this effect specifically to the AI-mediated word-selection process: since both experimental groups (AI-Visual and Real-Photo) outperformed the control group, the critical variable appears to be not the visual generation method but the computational selection of highly coherent words. This addresses the confounding concern by indicating that technology-mediated elements that shape the learning environment—CLIP and Word2Vec—may drive learning outcomes. However, this interpretation remains speculative given the quasi-experimental design, and causal claims are not warranted.

This result is consistent with the CALL literature and may extend to multimodal vocabulary teaching. Al-Seghayer (2001) found that dynamic multimodal annotations significantly improved second-language vocabulary acquisition compared to static presentations; Jones and Plass (2002) showed that integrated text-visual-audio explanations resulted in significant improvements in both comprehension and recall among French-language learners. Golonka et al. (2014), in a review of more than 350 technology-assisted language learning studies, found that evidence varies by technology type; however, interventions in which technology actively mediates the learning process, such as automatic speech recognition-based pronunciation training and chat-based interaction, receive the strongest empirical support. The current study adds to this evidence by showing that AI models embedded in the teaching system can also mediate vocabulary acquisition outcomes.

The high concreteness correlations (*r* = 0.93) reported by Kewenig et al. (2025) for multimodal transformer-based tools suggest that the computational selection method used in this study is well-grounded.

Particularly noteworthy is the absence of a significant difference in learning outcomes between AI-generated images and real photos. This aligns with the findings of Roca et al. (2024), who also reported that participants had difficulty distinguishing AI-generated images from real ones. This result is practical, as AI-generated images can provide more consistent visual support, eliminating variability and cultural bias in web-sourced photos. The low discrimination accuracy reported by Frank et al. (2024) in an international sample supports the use of AI-generated images rather than real photographs in educational contexts. However, as Nightingale and Farid (2022) point out, the ethical and copyright issues associated with AI images should not be overlooked. Crucially, this null finding should not be interpreted as evidence of equivalence between AI-generated and authentic photographs. The study was powered to detect only large effects (*d* ≥ 0.75), and smaller but educationally meaningful differences may have been missed. The non-significant difference reflects statistical uncertainty, not confirmed non-inferiority.

### 5.2 Mechanisms of Educational Psychology

#### 5.2.1 Self-regulated learning and vocabulary acquisition

The MSLQ findings indicated that the experimental groups reported higher intrinsic goal value, task value, self-efficacy, and cognitive strategy utilization compared to the control group. The positive correlation between intrinsic motivation and word acquisition enhancement (*r* = 0.48) supports the suggestion that highly adaptive AI-selected materials may reinforce learning not only through optimized content but also by enhancing learner engagement. This finding aligns with the theory of self-determination, which emphasizes the role of intrinsic motivation as a catalyst for deep learning (Ryan and Deci, 2000).

The high self-efficacy reported by the experimental groups may reflect the structured nature of the materials curated by CLIP, which offer clear visual-semantic maps that support successful vocabulary learning. According to Bandura’s social-cognitive theory, such mastery experiences are the most powerful source of self-efficacy beliefs, creating a positive feedback loop where success fuels self-confidence and greater effort. Nevertheless, because MSLQ data were collected only after the intervention, these group differences may represent preexisting learner characteristics rather than instructional effects. Causal attribution requires pretest-posttest designs with randomized assignment.

#### 5.2.2 Performance targets and learning patterns

The findings on achievement goals offer nuanced insights into how learners approach AI-mediated word tasks. The strong positive correlation between mastery-approach goals and vocabulary acquisition (*r* = 0.48) is consistent with theoretical predictions that learning-centered goals promote deep processing and sustainable engagement (Elliot and McGregor, 2001). In contrast, the negative relationship between performance-avoidance goals and acquisition (*r* = −0.24) suggests that fear of poor performance may weaken the exploratory interaction required for vocabulary acquisition.

These findings carry practical implications for AI-mediated learning design. Features that emphasize personal progression, skill development, and self-direction (mastery-approach framework) may be more effective than features that emphasize comparison or ranking with peers (performance-approach framework). The AI system’s ability to provide individual feedback and adaptive challenge can explicitly support the adoption of mastery-approach goals, shifting learners away from normative comparisons and focusing them on their own learning path. These are theoretically informed recommendations pending empirical validation.

#### 5.2.3 Cognitive load and multimodal design

The cognitive load findings support the thesis that well-designed multimodal instruction can optimize the allocation of working memory resources. The experimental groups reported lower unnecessary load and higher germane load compared to the control group; this suggests that the word-visual connections curated by CLIP redirect cognitive resources from unnecessary processing to efficient schema construction.

This pattern aligns with predictions of cognitive load theory, in which semantically aligned visual materials can benefit from the modality effect by distributing processing across visual and verbal channels (Sweller, 2011; Cepeda et al., 2006). The high visual-semantic alignment of experimental materials may have reduced learners’ need for intensive cross-modal integration, freeing up working memory capacity for deeper semantic processing.

However, the expertise reversal effect (Kalyuga, 2011) suggests that these benefits may be exclusive to beginners. As students develop greater proficiency and automation in word processing, additional visual support may become unnecessary. Future research should examine whether the cognitive load benefits of AI-selected materials persist or diminish at different proficiency levels.

#### 5.2.4 Basic psychological needs and motivation

The findings of the self-determination theory show that competence satisfaction is the strongest predictor of intrinsic motivation (*β* = 0.62), followed by autonomy (*β* = 0.31). This pattern supports the theoretical thesis that feelings of effectiveness and mastery are particularly critical in structured learning tasks such as vocabulary acquisition (Ryan and Deci, 2020).

The AI-mediated learning environment supports proficiency satisfaction through several mechanisms: (a) optimized word selection ensures learners encounter words at appropriate difficulty levels; (b) direct visual-semantic feedback confirms correct understanding; (c) Progressive difficulty structure builds cumulative mastery. Autonomy support can be enhanced through self-directed learning and personalized word suggestions; however, the current study did not directly manipulate these traits.

The non-significant unique contribution of relatedness may reflect the individual nature of the vocabulary acquisition tasks used. Future practices with collaboration features, peer interaction, or teacher-mediated social learning can enhance relationship satisfaction, thereby further strengthening motivation.

### 5.3 Stakeholder perceptions and technology adoption

The second-phase results indicated that students viewed the AI-powered model as somewhat motivating and fair, whereas teachers were more doubtful about its motivational impact. This observation concurs with Gavrilau et al.’s (2025) meta-analysis on the effectiveness of online testing, which found that students are generally open to technology-assisted testing, whereas teachers worry about control and predictability. Likewise, Rossic (2026) noted that English teachers showed a generally positive but cautious stance towards AI-powered tools.

Teachers’ view of the model as more challenging aligns with Cheng et al. (2004)‘s findings on performance-based testing. Alternative and tech-based assessments can add extra workload for teachers during planning, implementation, and evaluation. Li (2025)’s research on computerized writing grading systems indicated that teachers have mixed expectations—seeking efficiency but also concerned about quality. A similar dual perspective might be present here.

### 5.4 The relationship between fairness, motivation, and engagement

Correlation analyses showed a strong positive correlation between motivation and effort (*r* = 0.51) and a moderate correlation between perception of fairness and motivation (*r* = 0.42). These findings support the key predictions of the Technology Acceptance Model; perceived utility and fairness shape users’ attitudes and behavioral intentions towards technology (Davis, 1989; Davis and Venkatesh, 2004). The approach, based on the organizational justice theory of Colquitt et al. (2001), confirms that perceived fairness is a prerequisite for motivational engagement in this study. The study by McKinley and Rose (2026) in multicultural contexts demonstrated that cultural differences influence perceptions of technology. The diverse geographic origins of this study’s sample support the generalizability of the findings across cultural contexts.

### 5.5 Limitations and future research

This study has several limitations that need to be addressed transparently:

- Confounding Word Set variable: The most critical limitation is the use of different target words across conditions. The experimental groups received words with high CLIP, while the control group received words with low CLIP; This created a confounding variable that prevented us from attributing the observed differences solely to the teaching method. Characteristics specific to the words’ nature (frequency, tangibility, morphological complexity) may have triggered the observed differences. Future studies should either use the same word sets with different presentation methods (Option A) or match and control psycholinguistic variables (Option B).
- Absence of delayed testing: Without delayed assessment, outcomes are limited to immediate learning gains only. Large effect size (*d* = 1.42)—a large but defensible effect in the context of dense multimodal vocabulary (Al-Seghayer, 2001; Nation, 2001)—deserves careful interpretation. The brevity of the intervention may have magnified the effect, the novelty effect of the AI-mediated platform, the homogeneity of the starting sample, or the confounding variable of word set. To determine the robustness of this effect, repetition studies with longer-term, more varied proficiency levels and controlled word sets are required.
- Limited statistical power for image comparison: When n = 15 *per group*, the comparison between AI images and real photos is only sensitive to detecting large effects (*d* ≥ 0.75). Smaller but educationally significant differences may have been overlooked. The non-significant difference should not be interpreted as true equivalence.
- VKS measurement limitations: The Vocabulary Scale essentially covers its own perceived form knowledge rather than deep production capacity (Bruton, 2009; Waring, 1999). In future studies, the VKS should be supported by objective criteria such as Word Associates Format (Read, 2000) or controlled production tests (Laufer and Nation, 1999).
- CLIP language bias: CLIP’s training is predominantly English-focused, which can lead to bias in visual-semantic alignment scores for Turkish. The model may perform differently on culturally specific concepts or morphologically complex Turkish words. Developing multimodal models specific to Turkey can alleviate this limitation.
- Limited teacher sample: The 15 teachers in the second stage limit the generalizability of observational findings.
- TAM self-reporting limitation: The TAM questionnaire measures subjective perceptions without behavioral observation data; hence, it weakens causal claims about technology adoption.
- Evaluation of SRL and Goals Only Afterward: MSLQ and AGQ were administered only after the intervention; therefore, it was not possible to attribute the differences in SRL and goal orientation to instruction manipulation as causal. Pre-test measurements will strengthen internal validity.
- Methodological difficulties in vocabulary intervention design: Studies of vocabulary development face methodological challenges that affect the reliability of causal inferences. According to experimental design principles, only differences between variables were treated; therefore, only the teaching method needs to be changed so that each group learns the same target words (Al-Seghayer, 2001; Jones and Plass, 2002). The use of different word sets across circumstances introduces psycholinguistic variables such as frequency, length, concreteness, and morphological complexity; these factors can confound results and compromise internal validity. Deferred test evaluation is essential. Post-direct testing may reflect the effect of short-term recall or testing rather than continuous learning (Brown et al., 2008; Snoder, 2017). Without delayed assessment, researchers cannot distinguish between actual vocabulary and temporary performance improvement. Brown et al. (2008) showed that vocabulary decreases significantly over time and that retention periods indicate learning retention. Snoder (2017) emphasized the limitations of direct test designs, noting that a comprehensive curriculum with gradual retrieval promotes long-term memory.

Future studies may evaluate the potential of FastText or BERT-based models to process Turkish morphology more effectively (Bojanowski et al., 2017; Devlin et al., 2019). By developing Turkish-specific multimodal models, potential biases stemming from CLIP’s English-oriented training can be reduced. Furthermore, comprehensive frameworks that integrate video and audio modalities (Ionescu et al., 2025) can offer richer, more multimodal experiences in vocabulary instruction. Retention and transfer dimensions should be checked; interlingual studies should be conducted in languages with different morphological structures (e.g., Arabic, Hungarian, Finnish).

## 6 Conclusion and recommendations

This pilot study demonstrates that AI-powered multimodal concrete vocabulary instruction offers a potentially valuable framework, both analytically and empirically. Of course, all findings are preliminary and need to be replicated in more controlled settings. The results of the first stage reveal that high-scoring concrete words identified by CLIP and Word2Vec, combined with visual support, yield much better immediate learning outcomes than traditional methods. However, due to the confusion of word sets between conditions, it may not be correct to attribute these effects strictly to the teaching method.

Seeing AI-generated images as effective and consistent as real photos offers a practical solution to the variability problem in web-sourced images; however, this may reflect only limited statistical power rather than true equivalence. The non-significant difference between AI-generated and authentic photographs should be interpreted as the absence of evidence for a difference, not as evidence of absence of difference. From an educational psychology perspective, it appears that vocabulary instruction through AI supports self-regulated learning; this translates into higher intrinsic motivation, task value, and self-efficacy. In addition, adaptive achievement goal profiles (strong mastery-approach orientation), optimized cognitive load (reducing unnecessary load and increasing germane load), and the satisfaction of basic psychological needs (especially competence) play an important role in this process. These psychological mechanisms may partly explain the learning gains and illustrate important considerations in future AI-assisted instructional designs.

The findings of the second phase show that students develop a positive approach towards the AI-powered model; However, teachers are more cautious about motivational activities and the effort required. Perceived fairness and competence satisfaction play pivotal roles in motivating effort, and these elements should take center stage in the design of hybrid assessment strategies.

We can make a few suggestions to pedagogical educational institutions on these issues. First, instead of just looking at word frequency, it is helpful to consider computational visual-semantic fit metrics as well. Furthermore, AI-generated images can be evaluated for visual material preparation due to their consistency and accessibility. It is important to increase teachers’ digital assessment literacy; Professional development programs should be tailored to the pedagogical value of AI-powered tools. AI-assisted learning environments should be designed to support basic psychological needs—especially competence and autonomy. Additionally, instructional transformation should emphasize mastery-oriented goals, such as personal development and skill acquisition, rather than concerns about poor performance. To reduce cognitive load, it is important to optimize the load of multimodal presentations on working memory. Lastly, by developing hybrid assessment strategies, the reliability of traditional testing, the fairness of alternatives, and the accessibility of online testing can be achieved together.

In summary, AI-powered vocabulary teaching offers a data-driven and repeatable approach. At the same time, it takes into account stakeholders’ perceptions to ensure sustainability and continually validates them in the empirical field. It diligently addresses the methodological limitations identified in this pilot — through carefully designed repetition research using controlled word sets, delayed posttests, and objective word measures.

## Data Availability

The raw data supporting the conclusions of this article will be made available by the authors, without undue reservation.
